# The Meaning of “Hygiene” and Its Linked Practices in a Low-Income Urban Community in Bangladesh

**DOI:** 10.3390/ijerph19169823

**Published:** 2022-08-09

**Authors:** Rebeca Sultana, Nazmun Nahar, Nadia Ali Rimi, Sayeda Tasnuva Swarna, Shifat Khan, Md. Khaled Saifullah, Humayun Kabir, Peter Kjær Mackie Jensen

**Affiliations:** 1 Copenhagen Center for Disaster Research, Global Health Section, Department of Public Health, University of Copenhagen, 1353 Copenhagen, Denmark; 2 Institute of Health Economics, University of Dhaka, Dhaka 1000, Bangladesh; 3 icddr,b, Dhaka 1212, Bangladesh; 4 Department of Gastroenterology, Hepatology and Infectious Diseases, University Hospital Düsseldorf, Medical Faculty of Heinrich Heine University Düsseldorf, 40225 Düsseldorf, Germany

**Keywords:** water, hygiene, diarrhea, perception, Bangladesh

## Abstract

Improving hygiene practices is considered to be the single most cost-effective means of reducing the global health burden of infectious diseases. Hygiene promotion and disease prevention interventions often portray and promote “hygiene” from a biomedical perspective, which may not be optimally effective for achieving their goal of changing people’s behaviors. This study aimed to educe the meaning of hygiene for the residents of a low-income community in Bangladesh and how that meaning shapes their personal hygiene practices. We conducted this study in the Tongi township in Dhaka, Bangladesh, from September 2014 to June 2016. The research team purposively selected 24 households. The team conducted day-long observations using the participant observation approach and in-depth interviews with specific members of the 24 households. The concept of “hygiene” had two separate meanings to the study participants: cleanliness and holiness. The participants reported that cleanliness was required to remove odors, grease, hot spices and dirt. The motivation for cleanliness was to feel fresh, avoid heavy feelings, feel light and feel comfortable. To maintain the holiness of the body, bathing and ablution needed to be performed following particular religious rules/rituals. The motivation of holiness was derived from their accountability to God. The participants also reported that the cleansing processes and methods for the body and the home for cleanliness reasons were also different from those for holiness reasons. The notion of “hygiene” was multidimensional for the residents of the low-income urban community in Bangladesh. Our study participants did not explicitly conceptualize a notion of hygiene that was based on the germ theory of diseases but rather a notion that was based on individual physical comfort and cultural belief systems. Future studies on the prevention of hygiene-related diseases should combine and link the biomedical aspect to religious and cultural rituals to promote improved hygiene practices.

## 1. Introduction

Improving hygiene practices is considered to be the single most cost-effective means of reducing the global health burden of infectious diseases [1]. A number of efforts have been made to promote hygiene practices in the places where hygiene-related diseases are prevalent but have been unable to reach the expected success [2,3]. One of the important reasons for this limited success may be that disease prevention interventions mostly understand and promote “hygiene” from a biomedical perspective [4,5,6]. Hygiene promotion and disease prevention interventions often portray and promote “hygiene” from a biomedical perspective, which may not be optimally effective for achieving their goal of changing people’s behaviors. For example, the educational messages that promote handwashing mostly include the prevention of diseases and/or germs as a motivation, whereas the motivations of our study participants included attraction, affiliation, disgust, nurture and many more [7,8]. The germ theory states that microorganisms, which are known as pathogens or germs, are the causes of specific diseases [9].

Douglas in 1966 [10] and Curtis in 1998 [11] elicited important insights into the origins of hygiene, which are still considered as significant contributions to hygiene- and infectious disease-related research. A study on understanding the motivations for hygiene that was conducted in England in 2003 found that most household cleaning activities were motivated by the sight of dirt [12]. Another study that investigated the notion of hygiene found that it was an important social virtue for women as their hygiene practices were admired and judged by their peers [13]. In fact, “hygiene” is a social phenomenon and a social construction, which needs to be understood within the context of complex social realities to maximize the benefits of hygiene promotion interventions [11]. The meaning of and motivations for hygiene vary in different societies and settings. However, limited effort has been made to explore what shapes hygiene behaviors [8] and how individuals within different communities define the term “hygiene”. Furthermore, a recent review by White et al. (2020) suggested that studies that have explored the determinants of hygiene were suboptimal [8].

Hygiene promotion programs can be effective when they include the cultures and the social settings of the individuals who are the targets of the programs. Thus, understanding hygiene from local points of view (i.e., participants/consumers in different communities) could help to embed lay concepts and perspectives into the programs, which could then produce meaningful communication and promotion strategies for behavioral changes [11,13]. This could consequently help to develop culturally compelling behavior interventions to increase compliance with hygiene behavior. This study aimed to educe the meaning of hygiene among the residents of a low-income community in Bangladesh and how that meaning shaped their personal hygiene practices.

## 2. Methods

The detailed methodology of this study is provided elsewhere [14]; thus, we only include a brief description below.

### 2.1. Study Sites and Household Selection

We conducted this study in the East Arichpur area of the Tongi township in Dhaka, Bangladesh, from September 2014 to June 2016 [14]. East Arichpur is predominantly a low-income urban community with 13,876 households and approximately 55,504 people living within <1 km^2^ [14]. This study was a subcomponent of a larger longitudinal study (“Combating cholera caused by climate change”), in which 477 households were randomly selected to identify the risk factors for diarrhea [14]. To achieve our objective, the research team purposively selected 24 households from the 477 households that were involved with the larger study. First, we categorized the households based on water availability: 24-h availability and <24-h availability households. Afterward, within each group, we subdivided the households based on their water use methods: easy water use methods (e.g., running water from taps) and difficult water use methods (e.g., using a mug to pour water from water storage and/or using a hand pump). We considered these categories because we assumed that water usage varied based on the different methods and levels of availability. We also considered the availability and willingness of the participants to engage in this study as the presence of at least one member of the household was required to access the households for data collection. The detailed method for the selection of the 24 households has been provided elsewhere [14].

### 2.2. Data Collection

A team of anthropologists, including R.S., S.T.S., S.K., K.S. and H.K., collected data using an ethnographic approach. The first author provided hands-on training for the data collection tools that were used to observe the different phenomena of the individuals and the community. The team conducted community observations, household observations, in-depth interviews and informal discussions. The team conducted day-long observations using the participant observation approach and in-depth interviews with specific members of the 24 households. 

#### 2.2.1. Observations

Based on the availability and willingness of the participants to allow us to perform day-long observations, the team selected 12 households out of the original 24 to record their water usage for personal and domestic hygiene over the course of 24 h. The team conducted bimonthly observations for one year in those 12 households. The observations were from 12 to 14 h long (from early morning to night) and recorded the water use in each of the 12 households every other month. During the observations, the team measured the quantity of water that was used for each type of personal or domestic activity (e.g., washing hands, face, legs and genitals, bathing, washing clothes and dishes, cleaning the household and kitchen) by each member of the households [14]. The team did not observe some of the activities. The team asked the participants about how much water they used for bathing, urination and defecation after they performed each activity during the observations. The team recorded the amount and frequency of water that was used by all members of each household for personal hygiene. The team also recorded the amount and frequency of water that was used before performing religious prayers. To measure the volume of water, the team used a measured bucket and/or a measured mug before/after the activities. To measure the volume of water that was used for activities that required running water, the team used a stopwatch [14]. For example, we measured the capacity of the bucket that was used for bathing and after bathing, we collected information on how many buckets of water were used. For running water, we collected water from the tap in our measured bucket and recorded the time using a stopwatch to see how much water was collected in the bucket over a certain time period (e.g., 5 s, 10 s or 60 s). To attenuate any observation biases [15,16], the team spent 3–5 h in each household over a week before starting the observation sessions, which helped to desensitize the household to the researcher presence as outsiders. To attenuate any observer biases [16], the first author provided intensive training for all of the observers to develop the same understanding among the team members. 

#### 2.2.2. In-Depth Interviews

The team selected 36 participants from the 24 study households to conduct in-depth interviews, including 24 women and 12 men. Since women are considered to be responsible for maintaining domestic hygiene, water and sanitation within the household [17], we considered more women than men for the interviews to collect accurate data on domestic hygiene and water use. The household heads and their spouses were selected for the in-depth interviews. To conduct the in-depth interviews with males, we planned to select the household heads of the 12 observation households. The team conducted multiple sessions of in-depth interviews. The in-depth interview sessions were conducted in between the water usage observations. After one or two episodes of day-long observations in each household, the team conducted the in-depth interviews as the observations increased the rapport between the researchers and the participants. In the interviews, the team explored individual personal hygiene practices in terms of bathing, washing hands, face and legs and washing the genitals and anus after urination and defecation. The team also explored what the participants perceived about hygiene and how it was linked to water use practices in terms of frequency and quantity. The team also asked the participants whether they performed religious prayers in order to understand the differences between the perceptions and water use practices among participants who prayed regularly and those who did not. The different local terms used by the participants is included in the Table S1. The team also explored the reasons and rationales for water use by the participants for different personal hygiene activities. 

### 2.3. Data Analysis

All of the recorded audio data were transcribed verbatim in Bengali. The team then expanded all of the field notes regarding the observations and performed a thematic analysis [18]. R.S., S.T.S. and S.K. separately reviewed the transcribed data and expanded the field notes to identify any initial codes. After identifying the initial codes, a more comprehensive code list was developed, which considered the study objectives. R.S. reviewed and combined all of the coded data into different themes and subthemes to identify similarities and patterns, then summarized the findings. Later on, R.S. also made comparisons and triangulations among the findings from the observations, in-depth interviews and field notes from informal conversations since triangulation is important for the rigor of qualitative research [19]. 

To measure the water quantity that was used by the participants who performed religious prayers and those who did not, R.S. calculated the frequency, median and inter-quartile range (IQR) of the volume of water that was used for observed daily personal activities. 

### 2.4. Ethics

The data collection team obtained written informed consent from all of the participants who were included in this study or their guardians (for children). The ethical review committee of the icddr,b approved the study protocol.

## 3. Results

In total, 20 of the possible 24 in-depth interviews with women and 8 of the possible 12 in-depth interviews with men were conducted. The number of completed interviews was lower than planned due to the unavailability or unwillingness of the participants or the migration of the households to other communities. Most (81%) of the participants were aged between 18 and 45 years old, almost half (47%) of them earned an income and 22% of them were garment/factory workers (Table 1). During the observations, a total of 262 days of the activities of 59 participants (25 women and 34 men) were observed. A total of 24 of the participants who took part in the in-depth interviews reported performing religious prayers regularly: 17 out of the 24 women and 7 out of the 8 men. Out of all of the observed participants, 23 participants reported performing religious prayers regularly: 14 women and 9 men (5 women and 2 men were family members, not the in-depth interview participants). 

### 3.1. The Concept of Hygiene

**Figure 1:**
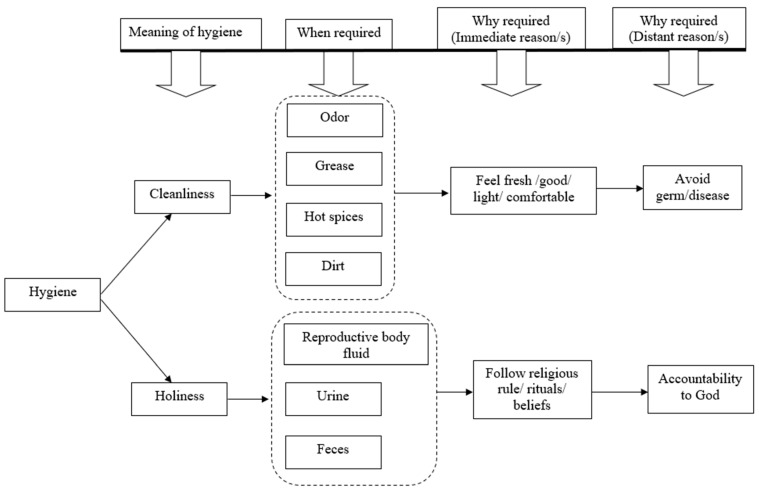
The concept of hygiene among the low-income urban residents of Arichpur, from September 2014 to June 2016.

The concept of “hygiene” had two separate meanings for the participants: cleanliness and holiness (Figure 1). The participants referred to cleanliness (“*parishkar-porichannata*” in Bengali) in terms of both personal cleanliness (cleanliness of the body) and domestic cleanliness (cleanliness of clothes, kitchen utensils, rooms, yard and sanitation facilities). The participants used the term holiness (“*pabitrata*” in Bengali) to refer mainly to cleaning the body, clothes and rooms. To maintain holiness, water was used to clean the body following particular religious rules/rituals. 

### 3.2. Cleanliness

Personal cleanliness included cleaning parts of the body (i.e., bathing and washing the face, hands, feet and genital areas) with only water and sometimes with water and soap/detergent (Table 2). The participants reported that the cleanliness of their sanitation facilities, clothing and kitchen utensils also required water and occasionally both soap/detergent and water. The cleanliness of their rooms did not require water all of the time but rather required keeping the bed and other furniture in the room tidy and in order. The participants reported that cleanliness was required to remove odors, grease, hot spices and dirt (e.g., any visible spots, dust, mud, etc.) (Figure 1). When we asked why cleanliness was required, the immediate reasons were to feel fresh, avoid heavy feelings, feel light and feel comfortable. After several probing questions, the participants occasionally mentioned avoiding germs/diseases as a reason for maintaining cleanliness (Table 2A).

There were two separate degrees of cleaning: one with water and the other with detergent/soap and water (Table 2B). Dust and other non-adhesive substances only required water while odors, grease and hot spices required both soap/detergent and water to ensure cleanliness. The participants also mentioned that any substances that were adhesive and/or stinky usually required soap as well as water (Table 2B).

### 3.3. Holiness

To maintain the holiness of the body, bathing and ablution were required for particular religious rules/rituals (Table 3). The participants said that for Muslims, ablution requires rinsing the face, hands, forearms, ears, nostrils, mouth and feet with water three times during each ritual. This was a mandatory practice that had to be performed before religious prayers. The other example was that clothes had to be washed with detergent and/or rinsed with water three times to ensure holiness. 

There were some particular events or activities that were considered unholy, which included contact with feces, urine, blood (including menstrual blood), fluid after childbirth and the body after sexual intercourse (Table 3A). Religious bathing (i.e., bathing while following religious rules, including reciting religious verses) was mandatory after menstruation and after sexual intercourse to return the body to a holy state (Table 3A). The participants also reported that they required more water to maintain the holiness of the body and the home. Those who performed regular prayers were more cautious about maintaining personal and domestic hygiene. 

Bodies that were smeared with urine or feces could only be cleaned with water to ensure holiness. To clean the anus and the genital areas while following religious rules for both men and women, the participants mentioned “*dhila kulukh*”, which is a small piece of cloth or a mud ball that is used to wipe the genital areas three times during each episode. The participants also mentioned that using soap was not required to wash hands when they used *dhila kulukh* as their hands did not touch the feces (Table 3B).

When we asked why holiness was required, the immediate reason was to follow religious rules (Table 3C). In response to more probing questions, the participants mentioned their accountability to God as another reason for maintaining holiness (Figure 1). 

**Figure 2:**
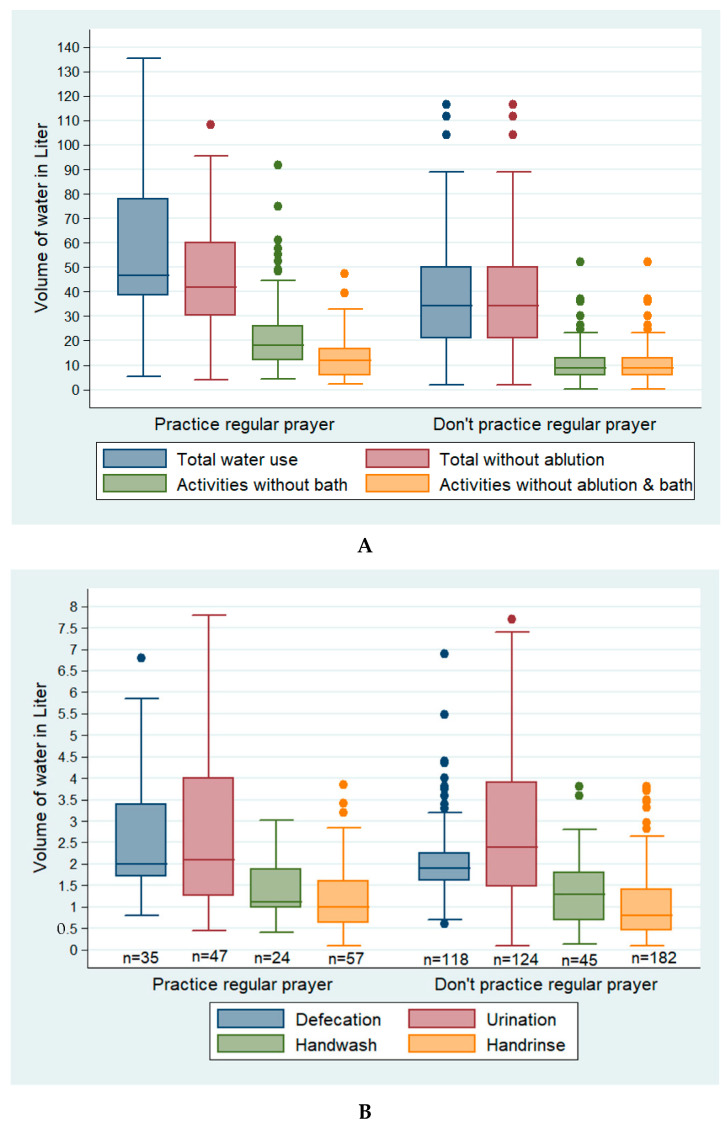
The differences between the water use of participants who practiced religious prayer regularly and the water use of those who did not practice religious prayers regularly among the low-income urban residents of Arichpur, Dhaka, from May 2015 to March 2016: (**A**) the personal hygiene practices of participants who prayed regularly (*n* = 61) and those who did not (*n* = 201); (**B**) the personal hygiene practices of participants who prayed regularly (*n* = 61) and those who did not (*n* = 201).

The participants who performed regular prayers used more water than those who did not perform regular prayers (Figure 2A). Skipping water use after urination was common among the men who did not perform religious prayers (13/22 men; skipped 33 times) compared to the men who performed religious prayers (1/9 men; skipped 3 times). Water use after defecation and hand rinsing (only using water for handwashing) was high among those who prayed regularly compared to those who did not (Figure 2B).

## 4. Discussion

The notion of “hygiene” was multidimensional for the residents of the low-income urban community in Bangladesh. Our study participants did not explicitly conceptualize/conceive a notion of hygiene that was based on the germ theory of diseases but rather a notion that was based on individual physical comfort and cultural beliefs (e.g., religious rituals and accountability to God). The risk of diseases or germs did not always influence their regular water use or hygiene practices.

Our findings had something in common with those of Curtis et al. [7], who found that comfort was one of the motivations for washing hands with soap and that it sometimes occurred as a part of religious rituals, which are a special form of habit. Similar to the study of Curtis et al. [7], our study also noted that being clean was a way of presenting yourself as pleasant and good in front of others and was an important motivator and indicator of status and affiliation, particularly among females. Curtis et al. [7] also reported that knowledge about the germ theory remained abstract among their study participants since germs are invisible and undetectable. Therefore, the findings of our study provided additional information in that comfort and religious rituals were not the only reasons for hand hygiene but were rather important factors in the general concept of “hygiene”, which included both personal and domestic hygiene. 

We found that participants who performed religious rituals and prayers used more water for personal hygiene compared to the participants who did not practice religious prayers regularly. Furthermore, skipping water use when cleansing the genitals after urination was another example of the male participants who did not abide by a belief system using less water. This suggested that gender roles could be a determinant of water usage and could contribute to the incidence of hygiene-related diseases as studies have suggested that the prevalence of diarrhea is higher among men than women [20,21]. In contrast, the female participants tended to use more water for specific activities that required shifting from an “unholy” state to a “holy” state (e.g., menstrual hygiene, religious bathing), which also helped to reduce the incidence of hygiene-related diseases. The use of more water has been linked to greater health benefits, as noted by Esrey et al. in 1991 [22,23]. Recently, Stelmach and Clasen (2015) conducted a systematic review of water quantity and health and found that increased water usage for personal and domestic hygiene was associated with a decreased incidence of diarrheal diseases [24]. Thus, it has been presumed that cultural belief systems may influence water use and improve hygiene practices.

Boyer and Liénard noted that cultural rituals are taught to individuals from childhood; thus, they become automated behaviors after extended periods of time [25]. We found that habits were important determinants of water use behaviors. For example, men avoiding the use of water after urination became a habit due to doing it for a long time. Foucault demonstrated that most elemental beliefs regarding health, family and right and wrong are not “natural” but rather nurtured over time through different institutions [26]. Thus, commercial campaigns for soap and detergent have become embedded into the cultural rituals of society over the course of time and have shown an opportunity for change. 

The ritual purification that was practiced by our study participants was not only required by Islam but also by other religions and belief systems, such as Hinduism and Christianity [27,28]. Participants from another study also reported cleanliness as being an important facet of religion [29]. The holy Koran states that the faithful should perform ablution (the ritual purity that is required for “*namaj*”) before the five daily prayers. The Laws of Manu, which is one of the four sacred Vedas of the Hindu scripture from circa 200 BC [27], states that “oily exudations, semen, blood, urine, feces, the mucous of the nose, ear wax, phlegm, tears, the rheum of the eyes and sweat are the twelve impurities of the body” and prescribes the avoidance of these 12 impurities. Cultural belief systems and the biomedical perspective both discourage contact with body fluids in order to maintain purity and hygiene. Although our study participants could perceive the concept of purity, they were not able to totally avoid contact with body fluids in practice. For example, the participants mentioned using the same cloth as a “*dhila kulukh*” (“*istinja*” in Arabic) repeatedly; however, in Islam, it is prohibited to use the same materials twice [30]. This recommendation might contribute to reducing the chances of contamination. However, our findings suggested that a gap existed between perception and practice and that some of the practices were culturally modified. 

Several studies have reported that biomedicine and germ theory have not been able to modify the beliefs of local people [7,31]. Explanations for disease causation that involve supernatural powers or God has been cited in several studies [31,32]. The advocation of causal explanations for infectious diseases using biomedical definitions to persuade communities to adopt preventive practices has often been missed in Bangladesh [31,32] as local communities are more convinced by traditional inherited knowledge, including religious norms, to define diseases and their prevention. The combination of local explanations and biomedical definitions to promote public health phenomena has been found to be effective in convincing local people to redefine disease causation and preventive practices [31]. Thus, building bridges between development and faith and science and spiritual approaches is vital to improve compliance with interventions. 

Different cultures and societies have different motivational drivers for adopting hygiene practices. Communities in England were found to be motivated to complete household cleaning activities by the sight of dirt [12]. Another study found that social virtue was an important factor for “hygiene” among women as their hygiene practices were admired and judged by their peers [13]. However, the complex associations between religion, culture and hygiene still remain essentially unexplored [32]. Therefore, our findings could be useful to guide future research on exploring whether cultural belief systems, including religion-oriented messages, could be effective in motivating people to improve their hygiene practices. 

Our study showed that the local understanding of hygiene was different from the biomedical understanding. We found that cleanliness for the sake of disease prevention was considered secondary and that cultural beliefs around holiness were rationalized as the primary reason for maintaining mandatory personal and domestic hygiene. Our participants compromised some hygiene practices that were linked to cleanliness but the practices that were linked to religion were considered compulsory. Thus, there is a need to alter methodological approaches to elucidate the local meanings of hygiene, determinants of hygiene and understandings of the social and moral aspects of hygiene to develop context-specific hygiene promotion interventions, which could be effective in preventing waterborne diseases, including diarrhea.

One of the limitations of this study was that we were only able to explore the perception of “hygiene” among Muslims as we did not have any participants of other faiths. We did not exclusively select Muslims on purpose; it was probably because the majority of the population in Bangladesh is Muslim. Thus, this study could not capture the diversity of perceptions of hygiene in terms of religious beliefs. Nevertheless, the concept of purity also exists in other religions [29]. Some of our findings might not be generalizable to the current worldwide scenario since we collected the data in 2016 and over the past two years, the concepts of hygiene and hygiene practices may have changed significantly due to the COVID-19 pandemic. However, cultural beliefs have not changed substantially in terms of the perception of hygiene and hygiene practices [33]. Although we tried to use a measured bucket and a stopwatch to obtain precise measurements of the volumes of water that were used, this method might not have been accurate in some cases. Since no standard method for water quantification exists for low-income settings [34], we only considered direct measurements to quantify the volumes of water. Nevertheless, our findings were similar to those from other studies that measured water quantities [35,36]. We found limited information on the use of cloths after defecation, so future studies should explore anal cleansing practices from the hygiene perspective in more detail. Furthermore, future studies should consider incorporating the perspectives of other religious groups to understand the social and health values of “hygiene” and compare them to the findings of this study. 

The meaning of “hygiene” is contextual. Biomedical researchers are interested in understanding the physical health benefits whereas our participants were interested in achieving spiritual benefits. Thus, a gap exists between the perceived benefits of hygiene practices among local people and the biomedical view of hygiene. Although there have been many studies that have described the differences between the hygiene practices of different genders including hand hygiene [37,38,39,40], more in-depth research on the differences between the personal hygiene practices of different genders should be undertaken in the future and should consider the relationships between gender-defined hygiene practices, the norms/reasons behind those practices and disease prevalence. Thus, interventions should focus on the contextual meaning of hygiene and tailor their motivational communication messages according to the context and society at which they are aimed in order to achieve the expected health benefits. Future studies on preventing hygiene-related diseases should combine and link the biomedical aspect with religious and cultural rituals to promote improved hygiene practices.

## Figures and Tables

**Table 1:** The participant profiles of the low-income urban residents of Arichpur, Dhaka, from May 2015 to March 2016.

Characteristics	Population (%)
Total Participants	32
***Gender***	
Male	8 (25)
Female	24 (75)
***Age***	
18–30	11 (34)
31–45	15 (47)
46–60	5 (16)
60+	1 (3)
***Religion***	
Muslim	32 (100)
***Non-Earning***	
Housewife	16 (50)
Unemployed	1 (3)
***Earning***	
Garment/Factory Worker	7 (22)
Small Business Employee	3 (9)
Service Holder	2 (6)
Day Laborer	2 (6)
Beggar	1 (3)
***Education***	
No Education	7 (22)
Did Not Complete Primary Education	7 (22)
Completed Primary Education	12 (38)
Completed Secondary Education	3 (9)
Completed Higher Secondary Education	1 (3)
Graduate	2 (6)

**Table 2:** Quotations regarding cleanliness from the study participants in Arichpur, Dhaka, from May 2015 to March 2016.

**A. Cleanliness of Body and Home**
“Suppose, I make myself, my room, and my bed clean. I wash all the utensils in my room; this is beautiful to me. I clean my body and I bathe using soap and water, put oil on my head, wash my clothes, these make me feel good. [What is the benefit of this cleaning?] The benefit is my body feels clean and I feel good. [Consider not washing yourself, what happens then?] I sweat during cooking. When my husband will ask for food, my child will ask for food, should I serve them with these hands? I wash myself, and I serve food. Then it feels clean and good to serve. [Is there any other reason for cleanliness?] No, not at all… Cleanliness is part of faith.” (A female participant.)
“Suppose, I dust off the room with a broom, pull ashes from the cooking stove, won’t it (hands and legs) get dirty? Then I will have to rinse my hands and feet. This type of dirty work requires rinsing of hands and feet.” (A female participant.)
**B. Cleanliness of Hands**
“To clean my cooking utensils, I scrub them with ashes, so black ash smears on my hands. Then I use soap to remove the black ash…soap helps to remove the stain. I use soap, because when my hands look black, it (ash/dirt) can smear on my face, cloth and eyes if I touch them, won’t it? … I use soap during bath to remove dirt and I feel good. If I cut fish, then I have to use soap as my hands stink. Suppose, you came and sat with me, you would say “move, you smell of fish”, won’t you say that? But if (I) wash my hands with soap it will not stink. Although nobody would get that stink (after defecation), it’s for my own sake and for my own conscience (I wash with soap after defecation). If I touch something or cut vegetables (without washing with soap after defecation), I feel bad myself thinking that, something can smear on my hand (after defecation), something can happen inside (my body). If I cook the food it can be spoilt, it can stink and if (someone) eats that food, his/her stomach can get upset.” (A female participant.)
“After cutting fish my hands get a fishy stench. After washing the cloth used for menstruation or after cleaning the child’s feces, it feels dirty and (I) feel uneasy that my hands are dirty. Nothing should be done or touched without washing those hands with soap. [Why, what happens if you touch anything (without washing)?] If I touch, I feel bad myself, there might be germ or (you know) dirt is dirt. It doesn’t feel good within me. That is why I wash my hands with soap.” (A female participant.)

**Table 3:** Quotations regarding holiness from the study participants in Arichpur, Dhaka, from May 2015 to March 2016.

**A. Activities That Were Considered Unholy**
“(After intercourse a bath is needed to be taken) This (bath) is separate (than normal bath). This has to be done within the (same) night. Body has to return to holiness, since (I) cannot touch anything with an unholy body, I cannot do any task. I do not even broom the floor. Whatever I do, I do it after bathing. The body is unholy after that (intercourse)…ablution is required separately (during bath) and religious verses have to be recited to make the body holy. Two to three times ablution is required (during bath). At first, I have to perform the ablution and then pour water on the body, and after completing the bath I need to perform ablution again to make the body holy. (During bathing, someone) can use soap, but it is not mandatory, using only water can make the body holy.” (A female participant.)
“During that time (religious bath after menstruation) I need two big buckets of water. I need to wash my hair, cut nails, clean genital properly otherwise, my prayer (*namaj*) will not be guaranteed… During that time, clothes need to be washed properly and thoroughly, three times (with water) otherwise it will not be holy. It (religious bath) has separate rules- starting with performing ablution, followed by pouring water down the whole body, then smearing soap on the body, again pouring water to clean the soap and finally perform ablution again… The body will become holy after the ablution. Regular bathing does not always require soap, so I do not use soap every day for a regular bath.” (A female participant.)
“So, my wife stays home from Thursday to Friday. She stays a bit unholy on Thursdays. During that time (intercourse), the room, for example, the bed and bedsheets remain impure/unclean/unholy. We change the bedsheets and put new sheets on, moreover, the bedsheets are washed on Friday. So, if Allah wants it (everything) will be holy then.” (A male participant.)
**B. Genital and Anal Cleansing**
“Usually when I go to the toilet, I do not use soap, but I do use a separate piece of cloth to clean myself. I use a cloth (wet the cloth with water) rather than using excess water. Yes, whether I defecate or urinate, I use cloth. After every two to three days I wash the cloth and dry it. Many people use soap but I usually just use the cloth” (A female participant.)
“Suppose cleanliness of prayer (*namaj*) includes using water *dhila kulukh* after urination. This way I became holy. Suppose I went to the toilet, washed my hands, this way I became clean and my body became holy. If I am not praying (*namaj*), it is okay if my body is unholy. If I am praying (*namaj*) 5 times a day, if I step on dirt or touch the chickens, I need to wash my hands with soap and then perform ablution. Moreover, if I have dirt on me (in fingers/nails), my prayer will not be accepted.” (A male participant.)
“Brother (referring to the interviewer), I do not use (after urination) water: there is no reason to it. Brother, you know it is the devils… It is because we Muslims are supposed to be pure and holy all the time. I do not even perform the regular prayers, so I do not even use water (after urination). Actually, it has become a habit, so I do not bother taking water (to use after urination).” (A male participant.)
**C. Religious Motivations For Cleansing Practices**
“I wipe the floor everyday as I perform prayer (*namaj*) every day at my house. Moreover, the floor and bed gets dirty with dust as children get on the floor with dust on their feet.” (A female participant.)
“Yes, after both defecating and urinating, I wash both hands, legs and then perform ablution. It is said to always stay in the state of holiness and purity by being in the state of ablution-because you never know when death will come. I can die anytime, even now, leaving all my work. That is why I stay in the ablution state, since dying while in the state of ablution will give me a lot of spiritual reward by the almighty.” (A female participant.)
“People perform ablution to stay holy and pure for regular prayer (*namaj*). If you can keep yourself always in ablution mode, it is good for you. Otherwise, people take bath only once a day, it is not possible to take bath five times a day. Therefore, ablution is the solution to remain holy. Moreover, if a Muslim man keeps himself always in holy state, it is good for him. No one can say when death will come. That is why, I think it is best to keep oneself in the holy state all the time.” (A male participant.)

## Data Availability

The data that are presented in this study are available upon request from the corresponding author. The data are not publicly available due to privacy issues. The information that was shared by the participants included personal and private activities/issues; thus, sharing the data publicly is not possible.

## Supplementary Materials

The following supporting information can be downloaded at: https://www.mdpi.com/article/10.3390/ijerph19169823/s1. Table S1. Different terminologies used in this manuscript.
