# Supplementary material for: The Meaning of “Hygiene” and Its Linked Practices in a Low-Income Urban Community in Bangladesh
Source: Int J Environ Res Public Health. 2022 Aug 9;19(16):9823. doi: 10.3390/ijerph19169823 (PMC9407852; doi:10.3390/ijerph19169823)
Supplement: Supplementary file 1 [file ijerph-19-09823-s001.zip › ijerph-1790853-supplementary.pdf]

Table S1: Different terminologies used in this manuscript

Personal hygiene	Cleaning of body parts including bathing, washing face, hands, feet, and genital areas, anus.
Domestic hygiene	Cleaning of cloth, kitchen utensils/dishes, kitchen space, rooms, yard and toilets, and bathrooms.
Rapport	A trust-building process to develop a harmonious relationship with community people and study participants to understand each other's feelings or ideas and communicate well.
Ablution	A mandatory practice Muslims should perform before religious prayers. It requires rinsing face, hands, forearms, ears, nostrils, mouth and feet with water three times during each episode.
Religious prayers (*namaj*)	*Namaj* is a ritual prayer prescribed by Islam, it has to be performed five times a day, and ablution before *namaj* is mandatory to ensure holiness
Unholy	Contact with feces and urine, blood and menstrual blood, fluid after childbirth, and body after sexual intercourse.
Holy state	The whole body is cleaned in a way which make sure that there is no body fluid or any other substance smeared anywhere in the body.
Religious bathing	A mandatory bathing and cleaning practices. In Islam, which is mandatory after the menstruation is over and after sexual intercourse.
'Dhila kulukh'	A small piece of cloth or mud ball to wipe the genital area for three times in each episode after defecation.
